# I don’t know what I am doing because I am doing everything: perceptions and experiences of nurses about HIV counselling and testing among children in Free State Province, South Africa

**DOI:** 10.1080/09540121.2016.1176670

**Published:** 2016-07-08

**Authors:** Abiola O. Olaleye, Yolisa Tsibolane, Lydia Van-Turha, Sibongile Monareng, Perpetual Chikobvu, Mohlouoa Sam Boleme, Celicia Serenata

**Affiliations:** ^a^ Clinton Health Access Initiative, South Africa; ^b^ School of Health Systems and Public Health, University of Pretoria, South Africa; ^c^ Free State Department of Health, HAST Division, South Africa; ^d^ Department of Community Health, University of Free State, Bloemfontein, South Africa

**Keywords:** HCT, counselling, testing, children, nurses, perceptions

## Abstract

Although HIV/AIDS constitute a significant health burden among children in South Africa, testing and counselling of exposed children are inadequate. It is therefore imperative that factors relating to paediatric HCT services offered by health workers are examined. This study was conducted to explore and describe the perceptions and experiences of trained professional nurses regarding HIV counselling and testing among children. We conducted six focus group discussions among trained professional nurses in health facilities in a district in Free State Province, South Africa. All verbatim transcripts were analysed with a thematic approach and emergent codes were applied. Forty-seven trained professional nurses participated in the study and two of them were males. The age of the participants ranges from 38 to 60 years while the median age was 50 years. Most participants in the focus groups explained how HCT occurs during regular health talks and that lay counsellors are doing most of the counselling. While a few participants thought that children should not be bothered with HCT, most of them seek consent from caregivers for HIV test for children. While children whose parents are negative are usually not tested, most children are tested only when they become ill. Identified barriers to HCT among children include refusal of consent, work overload, lack of encouragement, and poor record keeping. Participants recommended improvement of issues relating to community mobilization and increasing trained staff strength for optimal paediatric HCT service delivery. Developing guidance and policies with respect to obtaining consent, recruiting more health providers, and addressing structural issues in the society to reduce stigma and discrimination were identified as key priority issues by majority of the participants. The perspectives of these participants who provide paediatric HCT services offer vital insight which may be useful to inform policy interventions.

## Introduction

Although the rate of mother-to-child transmission of HIV has declined significantly in South Africa, 9200 new HIV infections had occurred among children in 2014 (UNAIDS, 2015). While initiation of antiretroviral therapy (ART) is recommended for all under-five children living with HIV for improved survival and quality of life (WHO, 2013), less than half of eligible children are receiving ART in South Africa (UNAIDS, 2015).

Studies have shown that gaps between children eligible for ART and those on treatment may be related to inadequate testing for HIV among those exposed (children whose mothers are HIV-positive) (Chhagan et al., 2011). While attitudes and practices of health providers are important in improving HIV counselling and testing (HCT) services (Yeap et al., 2010), there is paucity of studies in this regard. This study was therefore conducted to explore the perceptions and describe the experiences of trained professional nurses regarding HCT among children.

## Methods

HIV testing among children in South Africa is based on the principles set out in the Children’s Act - No. 38 of 2005, Children’s Amendment Act No. 30 of 2007, Sections 130–133 (McQuoid-Mason, 2007). Consent is often obtained for testing of infants whose HIV-positive mothers initiate antiretroviral prophylaxis for PMTCT. Furthermore, infants whose mothers were not part of a PMTCT programme, or who tested negative and are at risk of HIV infection and are breastfeeding are also tested for HIV. In addition, older children who attend hospital/clinic because they are ill and whose mothers’ HIV status is unknown, young adolescents (especially girls aged 11–15 years) who may be sexually active and at risk of acquiring HIV are also expected to be tested for HIV. To achieve universal coverage in ART services in the country, professional nurses were trained to provide comprehensive care to people living with HIV/AIDS including paediatric HCT using the Nurse Initiated and Maintenance of Antiretroviral Treatment (NIMART) strategy (Georgeu et al., 2012). Professional nurses are expected to counsel caregivers and/or children, obtain consent, collect blood samples and carry out HIV antibody tests, or send for HIV DNA Polymerase Chain Reaction (PCR) tests (for children younger than 18 months).

The present study was carried out in the Manguang metropolitan district of Free State Province of South Africa. The prevalence rates of HIV infection among children aged 0–4 and 5–14 years in this province were 1.7% and 2.7%, respectively (Shisana et al., 2014). It was conducted in Manguang municipality which has three sub-districts including Thaba Nchu, Botshabelo and Bloemfontein.

Focus Group Discussions (FGDs) led by the first author were carried out over a period of three days and professional nurses selected by purposive sampling participated in the study. Two sessions of FGDs were held with selected participants in each of the three sub-districts. The interview guide included open-ended questions about what happens in their respective health facilities as regards HCT among children; their experiences about paediatric HCT and; suggestions and recommendations for improved paediatric HCT services.

Transcribed audio recordings were compared with field notes. Data coding was done based on emerging topics and verbatim transcripts were analysed with a thematic approach. Data were analysed using Atlas.ti version 7 software (Atlas.ti, 2014). Ethical clearance was obtained from the Research Ethics Committee of the Department of Community Health, University of Free State, South Africa. Informed consent was obtained from all participants.

## Results

### Sociodemographic variables

**Figure 1.**
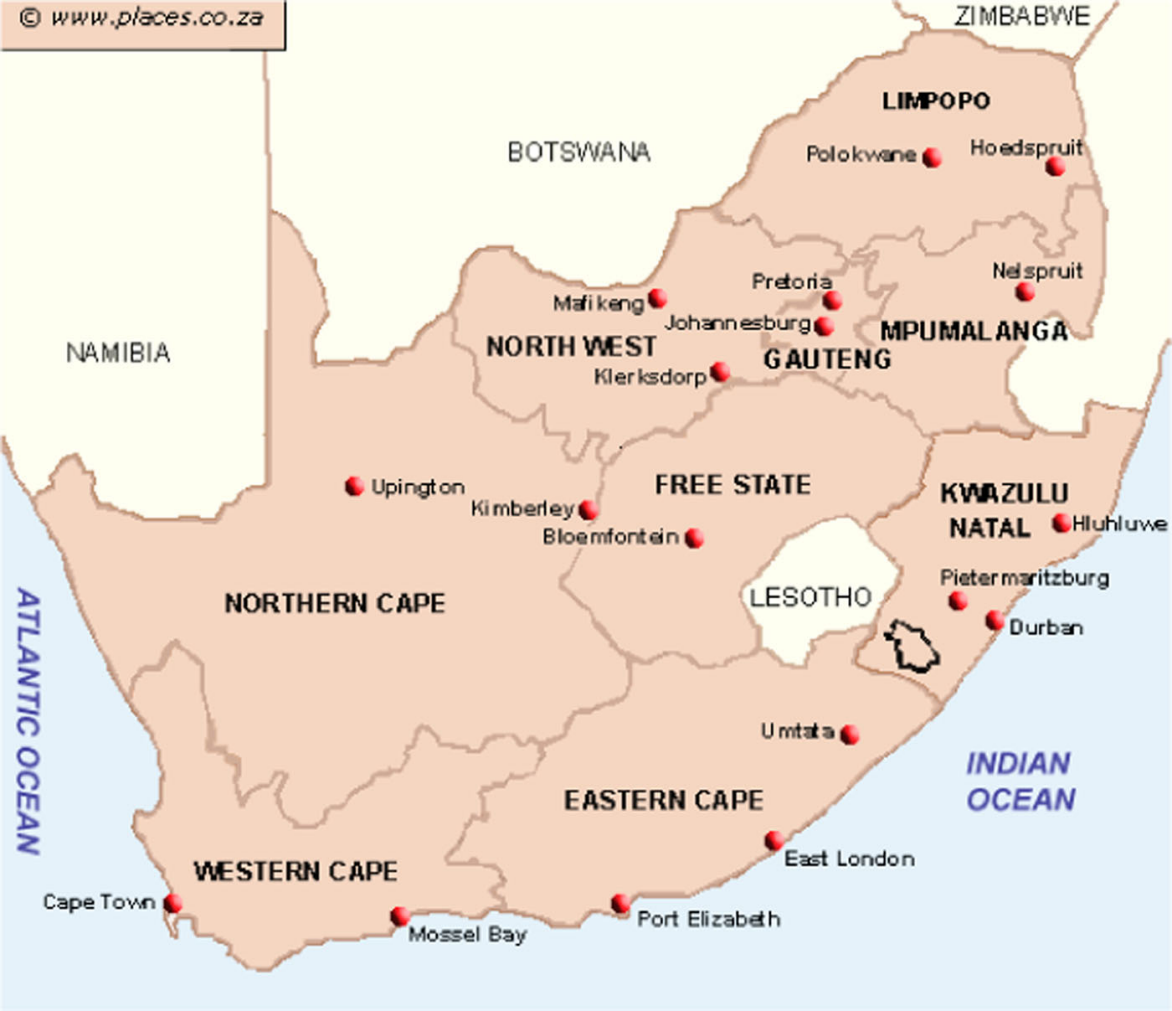
Map of Free State Province, South Africa (SA Places, 1997).

A total of 47 NIMART-trained professional nurses participated in the study. Two of them were males. The age of the participants ranges from 38 to 60 years while the median age was 50 years (Figure 1).

### Theme and categories

**Figure 2.**
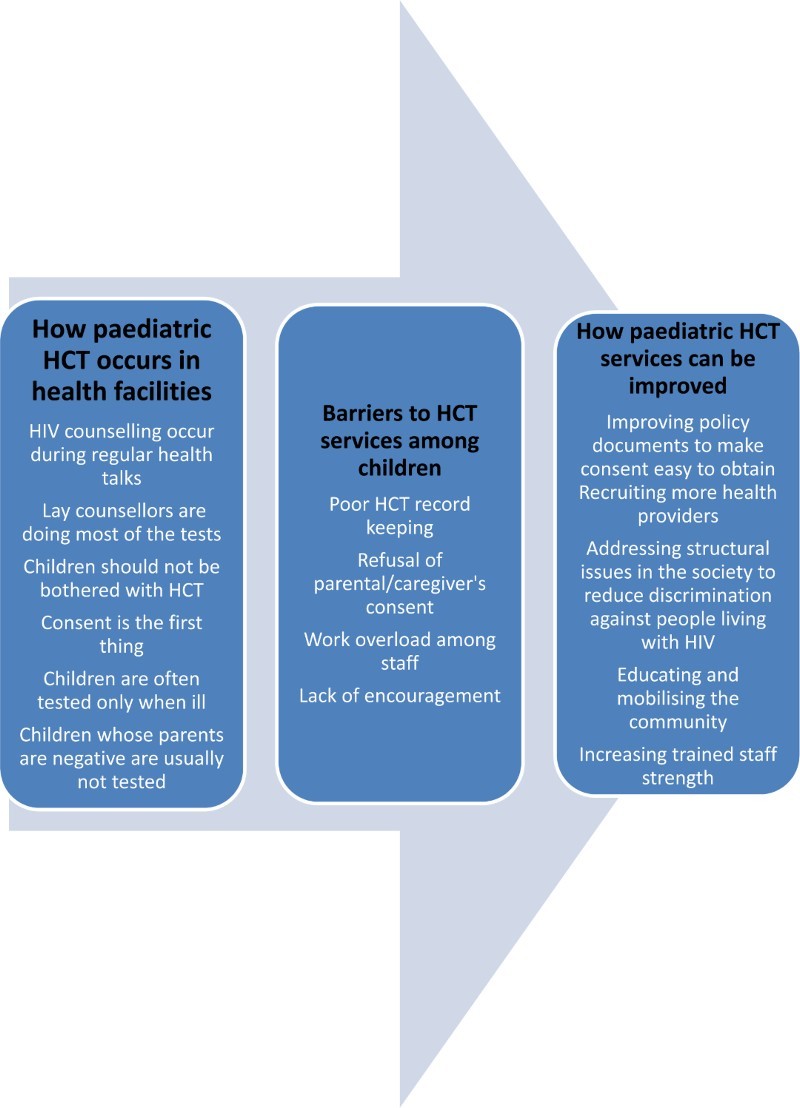
Perceptions and experiences of nurses among children.

**Figure 3.**
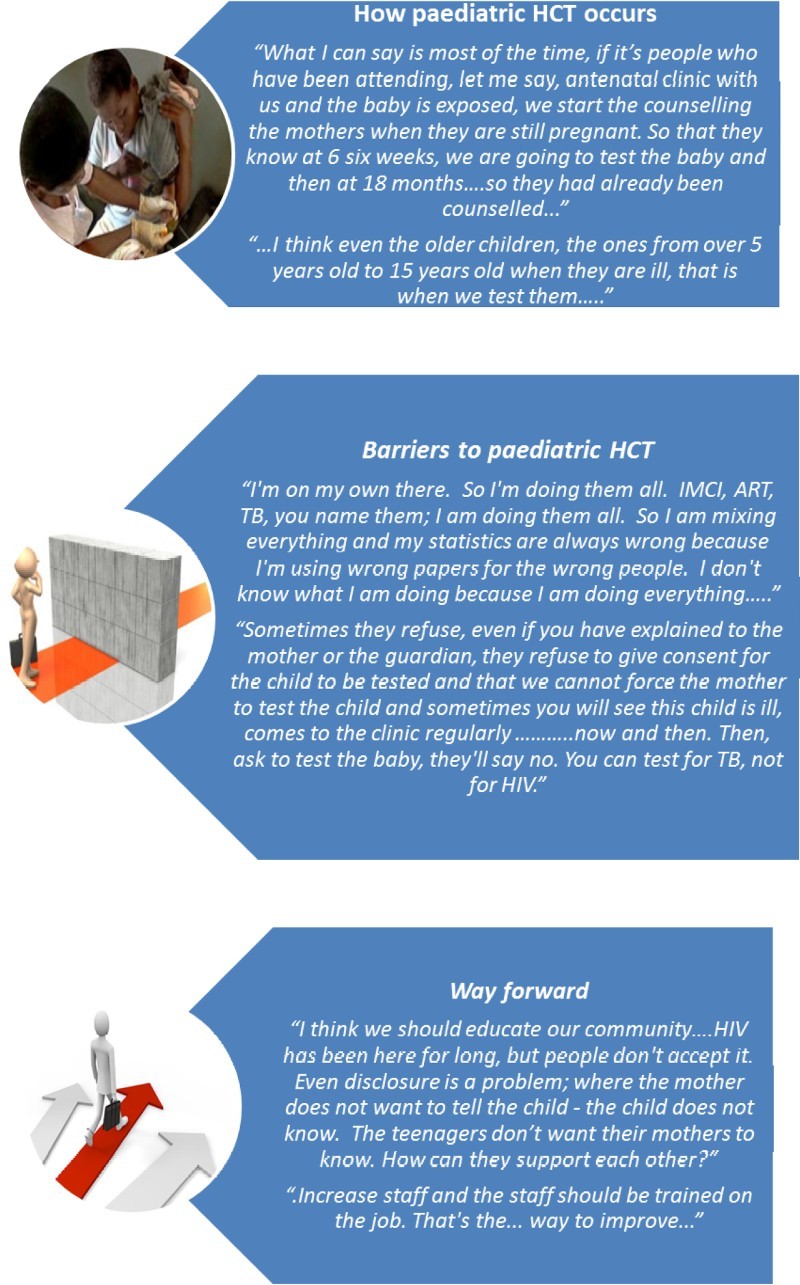
Some participants’ responses regarding paediatric HCT services (UNICEF, 2008; Goals and Achievements, 2016; ClipArtHut, 2016).

The following themes emerged from data analysis (Figures 2 and 3):

1 How HCT occurs among children in health facilities
  a HCT occur during regular health talk

Mass counselling for HIV is usually carried out during general health talk in antenatal and general outpatient clinics. This is usually done with all clients in the facility irrespective of the ages of the clients. Parents or guardians of exposed children are usually offered HCT. This was explained by a participant:

… whenever we are giving health education to the patients in the clinic, then we stress please can everybody be tested, from the new-born to adult.

Screening for tuberculosis is also another means of reaching out to clients regarding HIV testing in the health facilities.

… during TB screening, we usually enquire from the person who brought the child, was this child tested? So, if the child is not tested then we do test the child.

b HIV counsellors are doing most of the tests

Overall, HIV counsellors carry out majority of the HIV tests being done in clinics and hospitals in the study area. Thereafter, HIV-positive children are then referred to the professional nurses for continued care and treatment.

… So, the professional nurse has to test a certain amount of people in the consulting room, but most are coming from the HIV counsellor. So if counsellors counsel and test them, those that are positive, go through to the professionals and they take it from there.

c Children should not be bothered with HCT

Although parents and guardians of HIV-exposed children are the ones usually being counselled for HIV testing for their exposed children and wards, few participants think that children should not be bothered with HIV testing.

My problem with this whole issue of counselling to small children, do you know, a child up to 8 years cannot even think in abstract ways, so why would you over burden a child with information that he or she doesn’t understand and cause a lot of stress?

d Consent is the first thing

HIV counselling usually takes place in the facilities after obtaining consent from the parents/guardian of exposed children.

The first thing that is required from the mother is to get the permission to test the baby. When we have her consent, we do the testing.

e Children whose parents are negative are usually not tested

The participants expressed their opinions that it is unnecessary to test a child for HIV if the parents are negative.

I think, sometimes we think that because the parent tested negative that there is no issue and no reason to test the child … 

f Children are often tested when they are ill

Children are tested for HIV only when they are ill. Most of the participants believe that testing a seemingly healthy child is a waste of resources.

We test them when they are sick. Probably when you see this child is having pneumonia more than two times, or diarrhea, which we must look, or he’s losing weight or you are screening for TB, we also test for HIV.

Children who are five years and above usually do not come regularly to the clinics with their parents/guardians. Hence, most of them are usually not being tested in most health facilities.

For the children from 0 to 5 years they usually come to the clinic with their mothers, so testing is not something that is difficult. So for those from 5 to 14 years they are at school, they attend school, so they come only to the clinic when they are sick.

2 Barriers of HCT among children
  a Many negative HIV test results are not being reported

Participants reported that many negative HIV results are not being reported due to the excessive workload they have to handle.

Most of the counselling if the child is not positive most of the time because we are so busy and overworked, we may not remember reporting it. Because we have to write so much, sometimes we have tested the child and the child is negative but we have not recorded it on the history.

b Refusal of consent

Almost all participants emphasized the importance of seeking consent from the parent/care giver as the first step in HIV testing among children; refusal of consent was a major hindrance cited by most of the participants:

The problem that we are having with children who have a guardian they don’t always agree with the testing even if you see the symptoms and you tell the guardian or grandparents that it’s important for the child to be tested and screened … 

Parents/guardians often refuse to give consent for HIV testing even when their children/wards have suggestive symptoms.

They will tell you they are only here for immunisations, not any other thing; they don’t want to be manipulated on other things. They will say no, I am here for immunisations only.

Sometimes, guardians and caregivers may not know that some children are HIV exposed. Hence, they do not have adequate information to make a decision to allow an infant to be tested for HIV.

Sometimes it is difficult to do because sometimes these children are brought by a grandparent or somebody and then the person doesn’t know the status of the child and the child is due may be due for PCR.

c Work overload among staff

Professional nurses often struggle to cope with numerous work commitments. Sometimes, they are responsible for attending to patients who often present with myriad of health problems. A participant explained:

I’m on my own there. So I’m doing them all. IMCI, ART, TB, you name them; I am doing them all. So I am mixing everything and my statistics are always wrong because I’m using wrong papers for the wrong people. I don’t know what I am doing because I am doing everything … .

Another participant contributed to this:

… sister, there’s some, someone is delivering that side. You’ve got to leave everything there and go to deliver the baby on the other side. Oh, there’s an emergency and you leave everything and you go; every time you run; and at the end of the day, you are expected to do quality. And when you are trying to do quality, they want quantity. You are seeing your statistics, you say you are busy, but when you check on the statistics, you are seeing ten

Participants cited lack of adequate number of staff members as a challenge in HIV testing among children.

… Maybe there’s been one professional nurse there seeing the minor ailments, IMCI, the ARV clients and the chronics - one person is seeing those groups. So there’s no time for him to test the children … 

d Lack of encouragement

Participants expressed the perception that public health officials and supervisors who exercise oversight functions as regards their performances do not consider that they are hardworking. Rather, they assume that they are idle. This was expressed by a participant:

The sisters are sitting there doing nothing; they are sitting here, not knowing that we are busy with the patients. This is frustrating.

3 Recommendations for improved HCT
  a Doing away with consent issues

Most of the participants think that if the problem of obtaining consent from parents/guardians is simplified or removed, more children could then be tested. They also opined that the mode of seeking consent to test pregnant women for HIV should be adopted in the care of children. A participant explained:

I think we must do away with the consent. It must be done continually, as in pregnancy now … . If you can do it like that also, I think it will be better.

b Community education and mobilization

Education of the community through the mass media was proposed as a possible way of reducing stigma, and promoting confidentiality. Some participants also believe that issues relating to obtaining consent will improve if adequate health education can be carried out in the communities. One of the participants clarified her opinion thus:

I think we should educate our community … . HIV has been here for long, but people don’t accept it. Even disclosure is a problem; where the mother does not want to tell the child – the child does not know. The teenagers don’t want their mothers to know. How can they support each other?

Another participant also mentioned that health education is necessary to increase HIV testing among older children:

The other thing which can help us improve amongst the people in this age group (5–14 years) is health education … maybe during parents’ meetings, at churches; it should be stressed so that parents can know the status of their children … 

c Increasing trained staff strength

Increasing number and capacity of staff has also been cited by participants as a way of improving HIV tests among children:

Increase staff and the staff should be trained on the job. That’s the only way to improve.

There should be on-the-job training to improve the competencies of professional nurses in obtaining blood samples from children for HIV testing. This was explained by a participant:

… now they feel they are no longer competent in doing it, so they are scared to do it, so if there is a clinical facilitator, somebody that will teach, somebody who can come and demonstrate to us, you know, have like in-service training for certain procedures … 

## Discussion

This study described the perceptions and experiences of trained professional nurses regarding HCT among children in South Africa. It is unique, in that only few studies have explored the perceptions and experiences of health workers regarding HIV testing among children in literature (Kranzer et al., 2014), (Davies & Emma, 2014). This study found out that HIV counselling occurs regularly in the clinics and older children who are ill are often tested for HIV more than other children. Furthermore, refusal of consent and excessive staff workload were challenges against HCT among children while increasing trained staff strength and promoting community mobilization were cited as measures to improve paediatric HIV testing rates.

Participants in this study explained that HIV counselling occurs in their clinics during regular health talk. During antenatal period, pregnant women are often counselled for HIV testing. If a woman is found to be positive, she is then informed of the need to have PMTCT and HIV test for her infant at six weeks. This approach is similar to the Provider-Initiated Testing and Counselling among children described in other studies (Heunis et al., 2011), (Kranzer et al., 2014). Professional nurses and counsellors also offer HCT to clients accessing tuberculosis and other services. This optimizes the opportunities for HCT each time a client contacts the health facility. It is also in agreement with the practice described in a similar study in Namibia (Davyduke, Pietersen, Lowrance, Amwaama, & Taegtmeyer, 2015).

This study found that most HIV tests in the clinics are being done by HIV counsellors. This is similar to the findings of a qualitative study where a participant explained that it was unnecessary for her to carry out HCT for clients since there are HIV counsellors doing that (Kranzer et al., 2014). Although HIV counsellors are important in HCT services in health facilities (Mwisongo et al., 2015), leaving the services for counsellors alone may reduce the rate of HIV testing. This was shown by the findings of the Namibian study where the HIV testing rate was only 6.9% of the in- and out-patient encounters (Mwisongo et al., 2015).

The assumption that when children do not have symptoms of opportunistic infections, they may not have had HIV infection by vertical transmission. This may be responsible for the practice found in this study where older children are usually tested only when they are ill. While a similar practice was also reported in a previous study (Mwisongo et al., 2015), it is contrary to the recommendations of the WHO (WHO, 2010), and the South African national guidelines on HCT (Grant, Lazarus, Strode, van Rooyen, & Vujovic, 2012). Health workers should be sensitized towards recognizing the dangers of this assumption.

Excessive workload reported by nurses in the present study was also reported by a similar study among health workers in the Free State Province, South Africa (Heunis et al., 2011). This finding may be due the low ratio of health worker to population in most developing countries (Aluttis, Bishaw, & Frank, 2014).

Lack of encouragement from supervisors was cited as a major barrier to HCT services among exposed children in this study. This compares to the findings of a study which reported lack of infrastructure to encourage, monitor, and deliver HCT services as a barrier to HIV care services (Mwisongo et al., 2015). Similarly, 42.7% of health workers also reported that lack of adequate support from supervisors and unfair treatment were against good performance of their tasks in Iran (Daneshkohan et al., 2015).

Participants in this study suggested that issues regarding consent for counselling and testing have to be resolved to improve uptake of HCT services among exposed (children born to HIV-positive mothers) children. This is consistent with the findings of other studies where health providers cited difficulties encountered with counselling children for HIV test as a barrier to HCT uptake among children in South Africa (Davies & Emma, 2014).

Community mobilization cited in this study as a means of ensuring that more children are tested for HIV is consistent with the findings of a study in Sierra Leone where authors also reported community involvement and sensitization as a way of encouraging Voluntary Counselling and Testing (Anthony-Williams, Anthony, & Kanu, 2009).

## Conclusion

This study shows that health facilities often fail to provide adequate care to children due to difficulty in obtaining consent for paediatric HCT, and the challenges faced by healthcare providers in offering services to children. It is therefore important to develop clear guidance and policies to resolve issues relating to consent, recruit more healthcare providers, and improve provider-initiated counselling and testing among children. Efforts should be made to address structural issues regarding stigma and discrimination against people living with HIV in the society including children.
